# The Impact of Resource Availability on Bacterial Resistance to Phages in Soil

**DOI:** 10.1371/journal.pone.0123752

**Published:** 2015-04-09

**Authors:** Pedro Gómez, Jonathan Bennie, Kevin J. Gaston, Angus Buckling

**Affiliations:** 1 Biosciences, University of Exeter, Penryn, Cornwall TR10 9FE, United Kingdom; 2 Environment and Sustainability Institute, University of Exeter, Penryn, Cornwall TR10 9FE, United Kingdom; Graz University of Technology (TU Graz), AUSTRIA

## Abstract

Resource availability can affect the coevolutionary dynamics between host and parasites, shaping communities and hence ecosystem function. A key finding from theoretical and *in vitro* studies is that host resistance evolves to greater levels with increased resources, but the relevance to natural communities is less clear. We took two complementary approaches to investigate the effect of resource availability on the evolution of bacterial resistance to phages in soil. First, we measured the resistance and infectivity of natural communities of soil bacteria and phage in the presence and absence of nutrient-providing plants. Second, we followed the real-time coevolution between defined bacteria and phage populations with resource availability manipulated by the addition or not of an artificial plant root exudate. Increased resource availability resulted in increases in bacterial resistance to phages, but without a concomitant increase in phage infectivity. These results suggest that phages may have a reduced impact on the control of bacterial densities and community composition in stable, high resource environments.

## INTRODUCTION

Interactions between hosts and parasites can result in antagonistic coevolution, the reciprocal evolution of host defense and parasite counter-defense. Such coevolution can have important consequences for a range of ecological and evolutionary processes, including: population dynamics and extinction risk; the evolution of diversity and, ultimately, speciation; the evolution of sex and mutation rates; and the evolutionary ecology of pathogen virulence [1–4]. The dynamics, and hence consequences, of coevolution are highly dependent on environmental conditions [5], with resource availability likely to play a particularly important role [6,7]. However, while there have been a number of theoretical and laboratory-based empirical studies investigating the effect of resource availability on coevolution [6–16], experimental studies in natural environments are lacking. Here, we report results from two experimental approaches investigating the impact of nutrient availability on coevolution between bacteria and viruses in soil.

Theoretical and empirical studies suggest complex effects of resource availability on host-parasite antagonistic coevolution, but a general finding is that increased nutrients tend to result in the evolution of elevated host resistance [6,8–17]. There are two general reasons for this, which have proven hard to tease apart [15]. First, costs of resistance can be reduced when there is less competition for resources [18,19], allowing hosts to invest more in resistance and probably maintaining resistance even over periods of low parasite prevalence. Second, increased resource availability increases host population densities, and hence host-parasite encounter rates, promoting selection for resistance. As a consequence of increased host resistance with increased resource availability, selection for elevated parasite infectivity is likely in turn to increase, thus accelerating the rate of coevolution [6,15].

The relevance of these studies to natural populations is however unclear, as all the empirical studies to date (the majority of which involve bacteria and virus coevolutionary interactions) have been carried out *in vitro*. The one exception is a recent study of bacteria-virus (*Pseudomonas fluorescens* SBW25 and SBW25ø2) coevolution in soil, where the addition of nutrients in the form of in vitro growth media increased evolved levels of resistance to coevolving phage populations [17]. This increase in mean resistance occurred without a qualitative change in coevolutionary dynamics, which in soil is characterised by fluctuating selection: greater resistance to contemporary phage populations relative to those from a few generations in the past or future. Here, we experimentally investigate the generality of this finding under increasingly natural conditions by: 1) comparison of resistance of naturally occurring bacteria to their phage communities in the presence or absence of nutrient-providing plants; and 2) the addition of nutrients to experimental soil microcosms of *P*. *fluorescens* SBW25 and SBW25ø2 in the form of artificial root exudate. In addition to determining if this relationship between nutrient availability and resistance holds in natural systems, it is important to emphasise that dynamics of microbial communities in soil are crucial to terrestrial ecosystem functioning, and interactions between bacteria and viruses are likely to affect these dynamics [20–22].

## MATERIALS AND METHODS

### Natural bacteria-virus communities

As part of a larger experiment, closed mesocosms were established at the Penryn Campus (University of Exeter) in Cornwall, UK, comprising either plants of 18 grassland species (including 6 grasses, 6 leguminous forbs and 6 non-leguminous forbs) (high resource availability treatment); or no plants (low resource availability). One soil sample (~10 g) was collected monthly from 3 non-plant soils and 6 rhizosphere mesocosms, during 3 consecutive months. From each soil sample, 24 bacterial clones and a phage suspension were isolated on King’s media B (KB) agar as described in [23], providing for plant and no plant treatments, 18 and 9 phage suspensions and groups of 24 clones, respectively. Preliminary studies suggested very low phage infectivity levels, and hence phages from each replicate within treatments (plant or no plant) and time points were pooled, with resistance of each of the 27 bacterial communities (each containing 24 clones) tested against each of the two phage pooled phage communities. We tested susceptibility of each isolate to each of the two phage pools at each time point by spotting 35 μl of each phage community on each bacterial isolate growing in soft agar, with bacteria recorded as sensitive to phages when plaques were observed after overnight culture at 28°C. The data were then recorded as the proportion of natural bacterial isolates (out of 24 clones), that were susceptible to their plant-associated or non-plant associated phage pools.

### Real-time coevolution experiment

We carried out a fully factorial design between two 2-level factors (phage present or absent; and artificial root exudate added or not), resulting in 4 treatment combinations. *P*. *fluorescens* SBW25 strain marked for resistance to gentamicin [3] was grown overnight at 28°C in KB in an orbital shaker (180 rpm), and then centrifuged for 10 minutes at 3500 rpm to produce a bacterial pellet. This pellet was resuspended in M9 buffer to a final concentration of 10^8^ colony forming units (CFUs·ml^-1^) and 2.5 ml of this suspension inoculated into 24 replicate soil microcosms: 9 cm diameter petri dishes containing 30 g (unsieved) compost (John Innes no. 2). Microcosms were placed in an environmental chamber at 26°C and 80% relative humidity. Twelve microcosms were inoculated with the virulent bacteriophage SBW25Ø2 (10^6^ plaque forming units (PFUs) in 2.5ml M9) [24], and the other half with the same volume of M9 solution [20] to control for the addition of M9 per se. Half the microcosms in each phage treatment were supplemented with 5 ml of 2500 mg of labile Carbon (C)·ml^-1^ of synthetic plant root exudate solution [25], or 5ml of sterile distilled water to obtain soil microcosms under high- and low-productivity conditions.

Every 5 days for 20 days, soil samples (2 g) were collected using a sterile spatula and vortexed with 10 ml sterile M9 buffer and glass-beads for 1 minute. The resultant soil washes were diluted and plated onto KB agar supplemented with gentamicin (15 μg·ml^-1^KB) and incubated for 2 days at 28°C to determine CFUs per gram of soil. To isolate phages, a sample of each soil wash was vortexed with 10% chloroform and centrifuged at 13000 rpm. The phage supernatant was plated onto exponentially growing ancestral bacteria in 0.6% KB agar to enumerate PFUs [26]. From each replicate population and time point sampled, twelve bacterial clones and a phage suspension were stored at -20°C in glycerol solution (20%). Note that no culturable bacteria were detected that could grow on KB supplemented with gentamicin, nor could they be infected by phage SBW25ø2. Moreover, we did not find any phages in the soil wash that were able to infect *P*. *fluorescens* SBW25.

Twelve *P*. *fluorescens* clones from each population were assayed for resistance by streaking the bacteria against a line of phage (>10^3^ PFUs·ml^-1^) on KB agar: growth inhibition indicated sensitivity [15,24,26]. Experiments using ancestral phage demonstrated that this concentration of phage particles reliably inhibited the growth of sensitive ancestral bacteria. At each time point (except the last), bacteria clones were assayed for resistance against both the ancestral phage and their contemporary phage populations.

### Determination of synthetic solution concentration

We carried out a preliminary experiment to determine amounts of artificial root exudate to add to the soil microcosms to increase bacterial densities. The synthetic solution was prepared with 50 mM fructose, 50 mM glucose, 50 mM sucrose, 25 mM succinic acid, 25 mM malic acid, 12.5 mM arginine, 12.5 mM serine and 12.5 mM cysteine [25], resulting in 2.5 mg C·ml^-1^ and 150 μ*g* N·ml^-1^. Twelve soil-microcosms (4 treatments x 3 replicates) were inoculated with *P*. *fluorescens*, and then root exudate solution (5 ml) with different concentrations of labile carbon (0, 25, 250 and 2500 μg C·ml^-1^) applied every 5 days. Bacterial population densities after 20 days increased with increasing concentration of the root exudate solution (S1 Fig; *F*_3,8_ = 5.628, *P* < 0.023). We chose to use a concentration of 2500 μg C·ml^-1^ concentrations in the subsequent experiment, as the high-productivity condition since this led to a 10-fold increase in bacterial density.

### Statistical analyses

For the real-time coevolution experiment, analyses of the bacteria and phage densities, and bacterial resistance (square-root arcsin-transformed proportion) were performed by repeated measures linear mixed-effects models fitted by restricted maximum likelihood (REML); with time, the presence or absence of phages, nutrient addition, and ancestral and contemporary phages (and their interactions) fitted as fixed effects as appropriate, and replicate populations fitted as a random effect. For the natural community study, bacterial resistance (square-root arcsin-transformed proportion) was analysed as REML with bacteria source (plant/non-plant) and phage source fitted as two-level fixed effects, with replicate fitted as a random effect and time fitted as a fixed covariate. All analyses were carried out using JMP software.

## RESULTS

### Natural bacteria-virus communities

**Fig 1:**
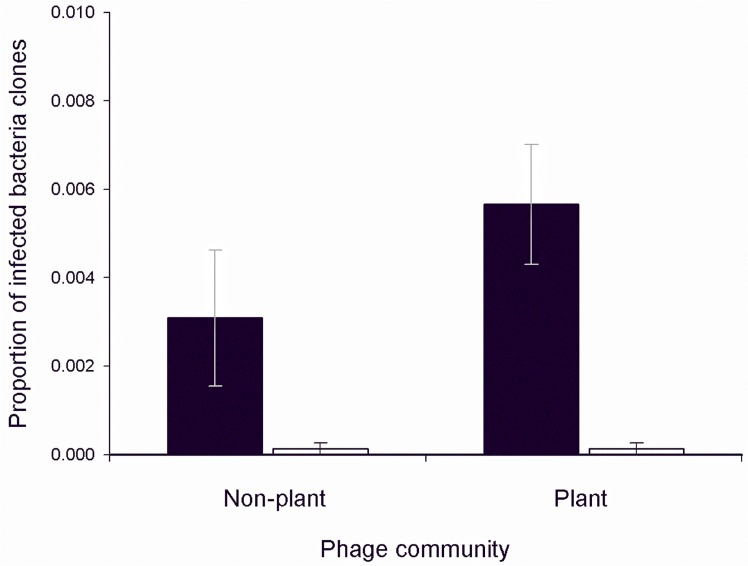
Proportion of infected bacteria clones. Bars represent the mean proportion (±SEM) of all bacteria clones isolated from non-plant associated (black bar) and plant-associated (white bar) soils that are susceptible to be infected by their non-plant and plant phage communities averaged across the three time points.

Bacteria isolated from plant-associated soils were more resistant (there was a greater proportion of resistant clones out of the 24 clones tested per community) to both plant associated and non-plant associated phage communities than bacteria that were not associated with plants (Fig 1; main bacteria effect against phage community; *F*_1,7_ = 8.11 *P* = 0.02). However, resistance did not differ against phages that were or were not associated with plants (*F*_1,7_ = 2.33 *P* = 0.17), and there was no interaction between bacteria and phage environment (Fig 1; interaction effect; *F*_1,7_ = 2.33 *P* = 0.17). In other words, plant-associated bacteria were more resistant, while the infectivity of phages was not influenced by their association or not with plants.

### Real-time coevolution experiment

**Fig 2:**
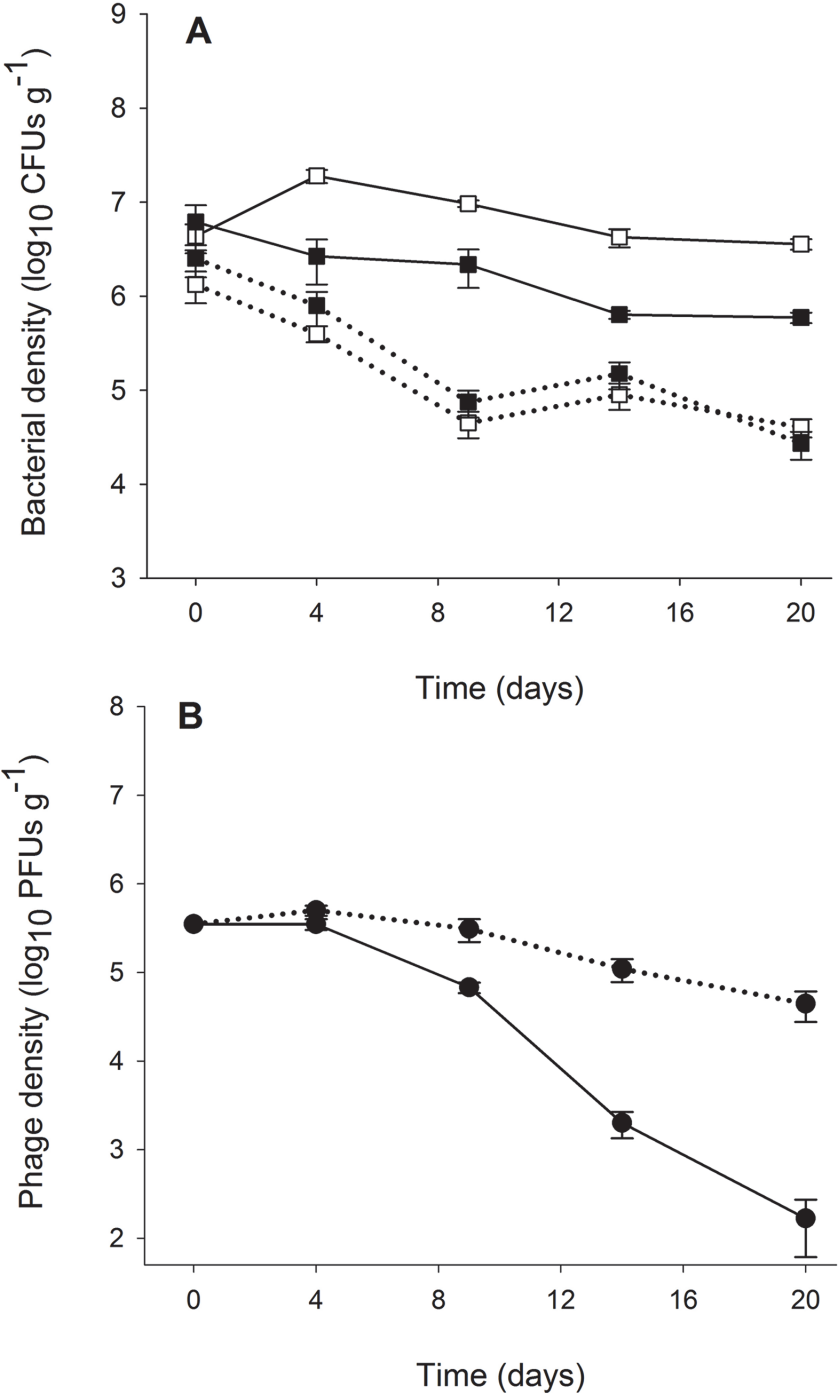
Bacterial and phage population dynamics in soil. Mean densities (±SEM) of (A) *P*. *fluorescens* SBW25 population (colony forming units·g^-1^ soil) in absence (□) and presence (■) of phages, and (B) phage SBW25ø2 population (plaque forming units·g^-1^ soil). Solid and dashed lines correspond to bacteria and phage population densities under nutrient addition or not, respectively.

The effect of phages on bacterial population densities was dependent on the addition of nutrients: phages had no net effect on *P*. *fluorescens* density in the absence of nutrients, but reduced density by approximately 10-fold when nutrients had been added (Fig 2; interaction effect, *F*_1,20_ = 13.15 *P* < 0.001). Considering only the phage treatments, nutrient addition increased *P*. *fluorescens* density by approximately 10-fold (main effect of nutrients; *F*_1,10_ = 10.76 *P* < 0.008), but dramatically reduced phage densities (main effect of nutrients; *F*_1,10_ = 29.33 *P* < 0.001), with phages driven to extinction in 4 out of the 6 replicates.

**Fig 3:**
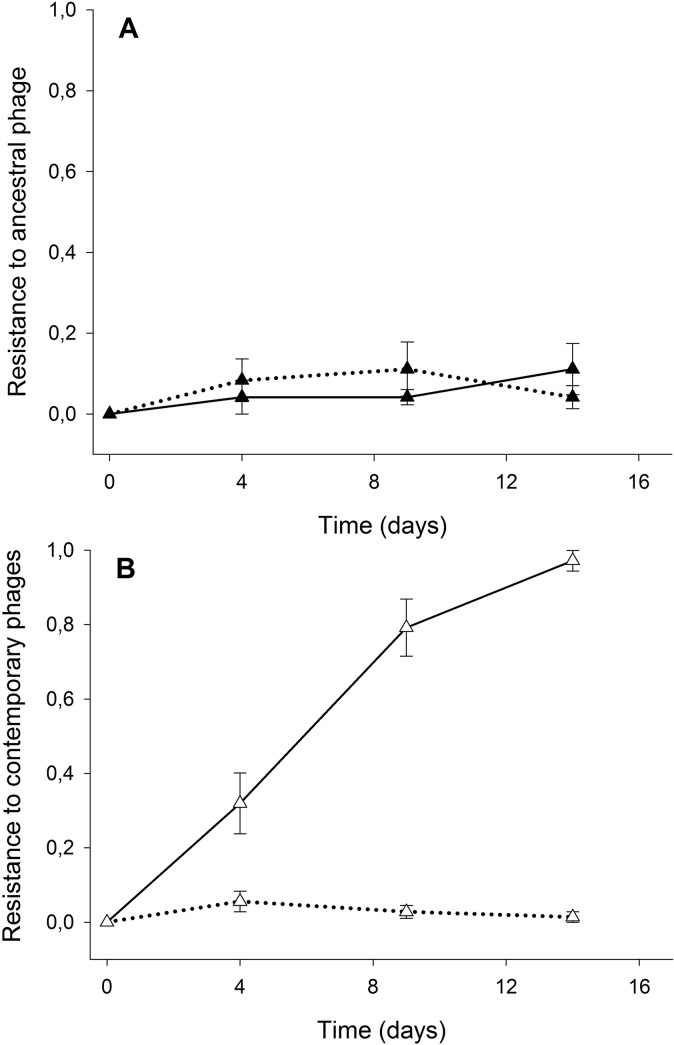
Rates of bacterial resistance evolution. Proportion (±SEM) of *P*. *fluorescens* SBW25 resistant to (A) ancestral and (B) contemporary phages through time. Solid and dashed lines correspond to bacteria resistance under nutrient addition or not, respectively.

Consistent with our previous work [17,20], we found that the frequency of bacteria resistant to the ancestral phage population was approximately 2% in both low- and high-productivity conditions (Fig 3A; low versus high productivity, *F*_1,10_ = 0.610, *P* = 0.45). However, bacterial resistance to contemporary phages was far higher with nutrient addition (Fig 3B; *F*_1,10_ = 116.16, *P* < 0.001). Resistance to contemporary phages was greater than to ancestral phages with nutrient addition (Fig 3; *F*_1,10_ = 178.64, *P* < 0.001), whereas resistance to contemporary and ancestral phages did not differ in the low nutrient treatment (Fig 3; *F*_1,10_ = 0.011, *P* = 0.92). Note that bacteria resistance was assayed at each time point, except the last, when phages became extinct.

## DISCUSSION

In this study, we investigated how resource availability affected bacterial resistance and phage infectivity in soil using two approaches that produced qualitatively consistent results. First, we measured resistance of bacterial communities to phage communities, and found this to be much greater for bacterial communities associated with plants. Phage infectivity did not differ between these communities. Second, we followed the real-time evolution of resistance and infectivity between initially clonal populations of bacteria and viruses in soil. Bacteria resistance evolved to be higher with nutrient addition, but without a concomitant increase in phage infectivity.

In the first experiment, the sampled bacteria and phages did not come from single species, and hence it is unclear if increased resource availability favoured more phage resistant species (species sorting), or more phage resistant genotypes within species (selection). Deep sequencing might in part address this problem, for example by showing that communities are the same or different under each treatment. Unfortunately, plant-associated and non-plant associated bacterial communities are known to differ in composition [27], hence knowledge of community composition would not help in assessing the relative importance of species sorting or selection. Moreover, it is unclear whether phages are directly driving patterns of bacterial resistance, or simply that high productivity favours bacteria that are coincidentally more resistant to phages.

The difficulty in interpreting the first experiment prompted us simultaneously to carry out the real-time experimental evolution study. In this case, the results unequivocally demonstrate that bacteria evolve greater resistance with increased availability of nutrients. However, as with the community data, phages were unable to increase their infectivity to the same extent as bacteria increased resistance, as reflected by the large increase in sympatric resistance (i.e. resistance to co-occurring phage) through time. Indeed, phages went extinct. The reason for this lack of increase in phage infectivity is very surprising, as a phage with ancestral-like infectivity profiles would have been able to infect sympatric bacteria, as reflected by the low levels of resistance against ancestral phages. These results are qualitatively the same when nutrient growth media (KB) was added to soil micrcosms [17]. It is possible that population sizes of the phages in this environment were simply too small, resulting in insufficient genetic variation. Note that there are not fundamental genetic constraints on the evolution of phage infectivity, as infectivity rapidly evolves *in vitro* [24,26].

It is likely that increased resistance was favoured with increased nutrient availability in part because of concomitant increases in bacterial density, and hence exposure to phages [15]. Resistance to phages is costly in soil [20], and increased exposure to phages would increase the selective advantage of resistance. Indeed, the density data show that increased resources markedly increased bacterial density, and moreover, phages had a much greater impact on reducing bacterial density under high resources. This latter result has been previously observed in both theoretical and empirical studies [6,8,9,15,28]. That this density reducing effect of phages appeared to persist for the duration of the experiment, even when resistance to contemporary phages was high and phages had been driven extinct, most likely reflects a time-lag for bacterial densities to recover.

Of concern here is that we did not find strong evidence of evolution of phage infectivity in the replicates without nutrient addition: there was no difference in the levels of evolved resistance to ancestral versus contemporary phages, suggesting that phages that had been evolving with bacteria in soil had the same infectivity profiles as the ancestral phage. This is in contrast with our previous studies [17,20] in which coevolution (i.e. reciprocal evolution of resistance and infectivity) was observed under similar conditions, as apparent by the higher resistance to contemporary phages than either past (ancestral) or future phage populations. One explanation may be that in this study soil microcosms were not mixed every time soil was sampled, as such population mixing can increase bacteria-phage encounter rates and selection for resistance and infectivity in soil [17] and spatially structured populations *in vitro* [29]. Alternatively, the soil used in the current study may have had fewer available nutrients than in the previous work (see below). There was, however, good evidence of coevolution with the addition of nutrients: bacteria evolved increased resistance to contemporary phage but little resistance to ancestral phages, demonstrating clearly that both bacteria and phages had changed with respect to their resistance and infectivity traits. However, coevolutionary dynamics were still characterised by fluctuating selection, as previously observed in soil [17,20], rather than an arms race dynamic, as is typically observed *in vitro* [24], where bacteria typically have higher levels of resistance against past than contemporary phages.

In summary, our results are consistent with theory and *in vitro* experiments in showing that high resource availability increases the density-reducing effects of parasites on hosts (i.e. the strength of parasite-imposed selection), and as a result, the evolution of host resistance to parasites. Moreover, increased host resistance in the current experiments was not associated with an increase in parasite infectivity. Our results suggest that high resource availability is likely to result in phages initially having a greater impact on bacterial densities in soil, but the resultant evolution of greater levels of resistance to co-occurring phages suggest that phages will subsequently have less of an impact in regulating bacterial communities. Indeed, this may help to explain why soil enrichment with plants or direct addition of nutrients can result in reduced bacterial diversity [30], assuming that phages play a role in maintaining bacterial diversity through frequency-dependent predation [31,32]. Moreover, the results have implications for the therapeutic and prophylactic use of phages to combat bacteria-associated disease of plants and animals, which are likely to be high resource environments for bacteria.

## Supporting Information

S1 Fig Effect of synthetic root exudate solution. Mean densities (±SEM) of *P*. *fluorescens* SBW25 (colony forming units·g^-1^ soil) at 20 days after inoculation, adding different concentrations of the artificial root exudates (0, 25, 250 and 2500 μg C·ml^-1^).
(TIF)
