# Supplementary figures and images for: The Impact of Resource Availability on Bacterial Resistance to Phages in Soil
Source: PLoS One. 2015 Apr 9;10(4):e0123752. doi: 10.1371/journal.pone.0123752 (PMC4391944; doi:10.1371/journal.pone.0123752)

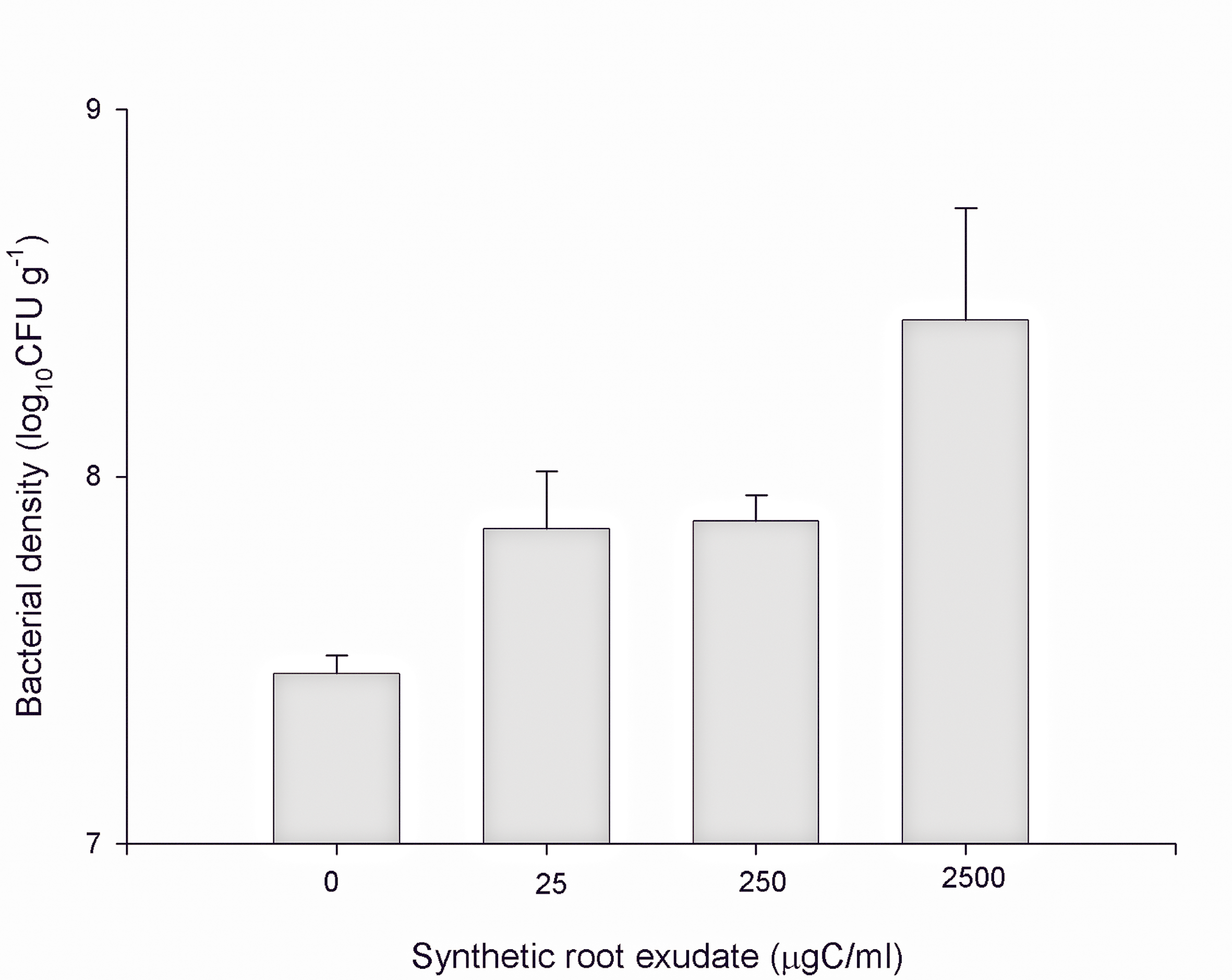

Supplement: S1 Fig — Mean densities (±SEM) of P. fluorescens SBW25 (colony forming units·g-1 soil) at 20 days after inoculation, adding different concentrations of the artificial root exudates (0, 25, 250 and 2500 μg C·ml-1). (TIF) [file pone.0123752.s001.tif]
